# Étude ethnobotanique des plantes utilisées dans le traitement du diabète dans la médecine traditionnelle de la région Maritime du Togo

**DOI:** 10.11604/pamj.2015.20.437.5660

**Published:** 2015-04-30

**Authors:** Gbekley Efui Holaly, Karou Damintoti Simplice, Gnoula Charlemagne, Agbodeka Kodjovi, Anani Kokou, Tchacondo Tchadjobo, Agbonon Amegnona, Batawila Komlan, Simpore Jacques

**Affiliations:** 1 Centre de Recherche et de Formation sur les Plantes Médicinales (CERFOPLAM), Université de Lomé, Togo; 2 Centre de Recherche Biomoléculaire Pietro Annigoni (CERBA), Ouagadougou, Burkina Faso

**Keywords:** Diabète, ethnopharmacologie, plantes médicinales, phytomédicaments, Togo, diabetes, ethnopharmacology, medicinal plants, herbal medicines, Togo

## Abstract

**Introduction:**

Les plantes constituent une grande source de principes actifs qui peuvent être utilisés pour traiter de nombreuses maladies, dont le diabète. L'objectif de cette étude était de recenser les plantes utilisées en médecine traditionnelle pour traiter le diabète dans la région Maritime du Togo.

**Méthodes:**

De janvier 2013 à juin 2014, une enquête ethnobotanique a été réalisée auprès de 164 guérisseurs traditionnels dans la région Maritime par des interviews directes à l'aide d'un questionnaire semi structuré.

**Résultats:**

Les données recueillies ont permis d'identifier 112 espèces végétales appartenant à 51 familles. Les familles les plus représentées ont été les Caesalpiniaceae / Fabaceae avec 9 espèces, suivie des Euphorbiaceae et des Compositae avec 8 espèces chacune. Les espèces les plus citées ont été Allium sativum, Alium cepa, Guilandina bonduc, Moringa oleifera et de Picralima nitida qui ont eu une valeur usuelle de 0,05. En termes de recettes, 132 recettes sont préparées à partir des 112 espèces de plantes. Les recettes à plantes uniques ont été au nombre de 78, tandis que 54 recettes sont obtenues par des associations de plantes. Les parties de plantes les plus utilisées ont été les feuilles suivies par les racines. La principale méthode de préparation reste la décoction.

**Conclusion:**

La région maritime du Togo dispose d'une biodiversité floristique importante en matière de plantes antidiabétiques. Ces résultats constituent une bonne base de données pour le criblage biologique dans la recherche de molécules antidiabétiques à base des plantes.

## Introduction

Un défi majeur de notre époque reste le traitement efficace et durable des maladies qui se sont accrues avec le fort taux d'urbanisation [1]. En 2011, lors de sa 66 ^ème^ Assemblée Générale, l'ONU a classé les maladies non transmissibles comme un nouveau défi dans la lutte pour l'amélioration de la santé [1, 2]. Au nombre de ces pathologies, le diabète prend une part prépondérante. Le diabète se définit comme une élévation anormale du taux de sucre dans le sang [3]. Il s'agit d'un désordre métabolique d’étiologie multiple caractérisé par une hyperglycémie chronique due à un défaut de sécrétion ou d'action de l'insuline ou les deux à la fois [3]. Selon la Fédération Internationale du Diabète (IDF), le taux de prévalence du diabète en Afrique subsaharienne sera augmenté de 98% entre 2010 et 2030 si des mesures urgentes préventives ne sont pas prises pour freiner son incidence. Au Togo, le diabète est en pleine expansion, du fait du changement de mode de vie de la population. D'après les résultats des enquêtes réalisées par le Ministère de la Santé en 2010 et en 2012, la prévalence du diabète est de 2,6% au sein de la population âgée de 15-64 ans. L'utilisation des plantes pour se soigner est une question de culture et de tradition en Afrique. Il est à noter que pour les besoins de santé primaire, une grande frange de la population africaine a recours à la médecine traditionnelle, dont les remèdes sont essentiellement à base de plantes [4, 5]. Comme pour les autres pathologies, les cas de diabète sont aussi pris en charge en médecine traditionnelle. La particularité du diabète est qu'il a une prise en charge très contraignante dans la médecine moderne, notamment la prise régulière de la glycémie et l'injection journalière de l'insuline. Outre ces contraintes, les moyens financiers pour le suivi conduisent les populations des pays en développement à se tourner définitivement vers la médecine traditionnelle pour la prise en charge du diabète [6]. De plus en plus, les études scientifiques se focalisent sur l'utilisation des plantes dans le traitement du diabète par la médecine traditionnelle à travers les enquêtes ethnobotaniques et les criblages biologiques au laboratoire sur les modèles animaux [7, 8]. Cependant, les données scientifiques concernant le traitement du diabète en médecine traditionnelle sont insuffisantes. Au Togo, une étude ethnobotanique menée dans la région centrale du pays a donné une documentation sur l'utilisation des plantes dans le traitement du diabète par les populations indigènes mais les données sur leur efficacité sont quasi inexistantes [4]. La présente étude a été initiée en vue de recenser les plantes utilisées dans la région maritime du Togo dans le traitement du diabète.

## Méthodes

**Cadre géographique d’étude**: le Togo est un pays de l'Afrique de l'Ouest, limité au Nord par la République du Burkina Faso, à l'Est par la République du Bénin, à l'Ouest par la République du Ghana et au Sud par l'océan Atlantique. Le Togo est divisé du Nord au Sud en cinq régions économiques: la région des Savanes, région de la Kara, région centrale, la région des Plateaux et la région Maritime. La présente étude a été réalisée dans la région Maritime. Cette région s’étend entre 1° 20’ de longitude Ouest et 1° 50’ de longitude Est et entre 6° 10’ de latitude Sud et 6° 60’ de latitude Nord, sur une superficie de 6100 km2, soit environ 10,78% de la superficie totale du Togo. La région Maritime est bordée au nord par la Région des Plateaux, à l'ouest par la République du Ghana, à l'Est par la République du Bénin et au sud par l'océan Atlantique. Le climat est subéquatorial avec une longue saison des pluies de Mars à Juillet (maximum de 1200 mm en Juin) et une courte saison des pluies de Septembre à Novembre (maximum de 1000 mm en Octobre). Les précipitations minimales pour les deux saisons sont respectivement de 184,4mm et 6,9mm. La température annuelle moyenne est d'environ 27,5 °C avec un maximum autour de 35,1 °C pendant la saison sèche. La région est fortement dégradée, la végétation est composée des forêts disparates, des reliques de forêts galeries, savanes, prairies, fourrés littoraux ou marécageux halophile. Les données démographiques de la région ont révélé une densité de 100 à 200 habitants par km2 pour les zones de fortes densités, et 50-100 pour les zones de plus faibles densités. La région est habitée par 1.828.000 personnes, les principaux groupes ethniques étant les Ewe, les Ouatchi et les Mina.

**La collette des données**: l'enquête a été effectuée chez les tradipraticiens de la région à l'aide d'une fiche d'enquête. Un premier entretien a été effectué avec les tradipraticiens pour leur donner une explication succincte des objectifs de l’étude et de l'importance des renseignements qu'ils allaient fournir, afin d'obtenir leur consentement à participer à l’étude. La collecte des données a été ensuite réalisée grâce à des interviews suivant un questionnaire semi structuré rédigé pour la circonstance. Le questionnaire a été axé sur les principaux points suivants: i) l'identité de l'enquêté: nom, prénoms, âge et sexe; ii) l'origine du savoir: initiation au sein de la famille ou dans un autre cadre, iii) le statut du guérisseur: guérisseur à temps plein ou à temps partiel, iv) la maladie: nom de la maladie dans la langue locale, les symptômes qui aident à poser le diagnostic, v) les plantes utilisées dans le traitement de la maladie, les organes de plantes utilisée, le mode de préparation de recettes et l'administration. Après les interviews avec les tradipraticiens, les échantillons de plantes ont été collectés et des photographies ont été prises sur le site pour aider à l'identification des plantes. L'identification des plantes a été effectuée au laboratoire de botanique de la Faculté des Sciences de l'Université de Lomé par comparaison avec les spécimens disponibles dans l'herbier de ladite faculté et ou de l'herbier national si nécessaire. La taxonomie a été confirmée en s'appuyant sur les données disponibles sur le site du « International Plant Names Index » (IPNI):http://www.ipni.org/.

**Analyses des données**: les données recueillies à la suite des enquêtes ont été traitées en utilisant le logiciel tableur Excel 2007 qui, a permis d’établir les fréquences d'utilisation des espèces et leurs valeurs usuelles (VU) suivant la formule ci-après. VU= ΣN/n Où ΣN = nombre de fois que l'espèce est citée dans les recettes et n = nombre de personnes enquêtées

## Résultats

**Données sociodémographiques des tradipraticiens**: la présente étude a enrôlé 164 tradipraticiens (TD) de la région Maritime du Togo dont 133 de sexe masculin et 31 de sexe féminin. Leur âge moyen a été 52,26± 15,77 ans avec un minimum de 20 ans et un maximum de 98 ans. Le Tableau 1 résume les données sociodémographiques de ces TD. Les tradipraticiens ont été répartis en 5 classes d’âges. L'analyse du Tableau 1 montre que la majorité des TD se retrouvent dans les deux classes d’âge de 30 à 50 et de 50 à 70 ans. Ces deux classes d’âges regroupent à elles seules plus de 75% des enquêtés. Environ 80% de ces TD sont scolarisés, 18,29% ont atteint le niveau secondaire et 8,54% le niveau universitaire. Pour ce qui est de l'origine de leur savoir, la majorité soit 64,63% ont été initiés à la pratique de la médecine traditionnelle au sein de la famille, tandis que les autres ont été initiés en dehors du cadre familial. Les résultats de l'enquête ont aussi révélé que 42,07% des TD exercent la médecine traditionnelle en plein temps, tandis que les autres la pratiquent en activité secondaire. Pour ce qui est de la saison et le moment de la récolte des organes de plantes, les données ont aussi varié en fonction des TD. Certains préfèrent récolter le matériel végétal en saison sèche et d'autre en saison des pluies, mais le facteur qui joue le plus c'est la disponibilité des plantes en ce qui concerne les plantes saisonnières.

**Tableau 1:** Données socio démographique des tradipraticiens de la région maritime

Sexe	Masculin		Féminin		
N	133		31		
%	81,10		18,90		
**Tranches d’âge**	**< 30 ans**	**]30-50 ans]**	**]50-70 ans]**	**] 70-90 ans]**	**>90ans**
N	35	68	60	20	3
%	7,93	41,46	36,58	12,19	1,83
**Scolarisation**	**Analphabètes**	**Primaire**	**Secondaire**	**Universitaire**	
N	33	87	30	14	
%	20,12	53,05	18,29	8,54	
**Origines du savoir**	**Héritage familial exclusif**	**Révélation divine**	**Initiation traditionnelle**	**Autres**	
N	106	30	26	2	
%	64,63	18,29	15,86	1,22	
**Statut du guérisseur**	**A plein temps**	**éleveurs et Agriculteurs**	**Secteur formel**	**Artisans**	
N	69	26	52	17	
%	42,07	15,85	31,71	10,37	
**Saison de la cueillette**	**Sèche**	**Pluvieuse**	**Toutes les saisons**		
N	14	58	92		
%	08.20	35,55	56.25		
**Moment de la journée**	**Matin**	**Midi**	**Soir**	**Toute la journée**	
N	74	4	14	72	
%	45.31	02.73	08.20	43.76	

Les données dans le tableau sont exprimées en effectif (N) et en pourcentage (%)

**Moyen de diagnostic du diabète par les tradipraticiens**: le diagnostic du diabète par les tradipraticiens repose uniquement sur les symptômes. A cet effet, plusieurs symptômes ont été cités. Parmi ces symptômes, la polyurie, les urines visqueuses ou mousseuses, la difficulté de cicatrisation et les vertiges sont les plus représentatifs, cités respectivement par 39,02%, 31,71%, 26,22% et 24,40% des répondants. D'autres symptômes comme les vomissements après prise de médicament, le noircissement du sang, les ictères et les ulcérations internes ont été moins cités (Tableau 2). Ces symptômes cités sont liés au cas de complications du diabète sucré évoluant vers une altération chronique des fonctions de certains organes. L'enquête a montré que les tradipraticiens de la région maritime ont des notions sur les symptômes qui définissent la maladie.

**Tableau 2:** Symptômes des diabètes tels que cités par les tradipraticiens

Symptômes	% Répondants (n = 164)
Polyurie	39,02
Urines visqueuses ou mousseuses	31,71
Difficulté de cicatrisation	26,22
Vertiges	24,40
Urines sucrées (attraction des fourmis)	19,51
Œdèmes	18,29
Asthénie	17,07
Faiblesses sexuelles	12,20
Douleurs à la miction	12,20
Amaigrissement	10,98
Urines nauséabondes	9,15
Fièvre	9,76
Céphalées	9,76
Difficultés respiratoires	9,15
Polydipsie	9,15
Vomissements après prise de médicament	8,54
Noircissement du sang	6,10
Ictère	4,88
Ulcérations internes	2,44

Les données dans le tableau representent les pourcentages des tradipraticiens citant un symptôme pour un total de 164 répondants

**Figure 1:**
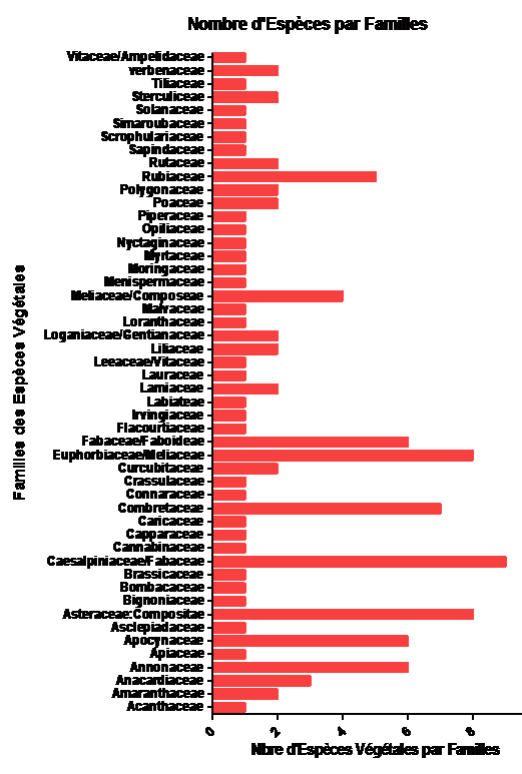
Répartition des espèces végétales au sein des familles

**Caractéristiques botaniques et diversité des plantes à propriétés antidiabétiques**: au total 112 espèces végétales ont été répertoriées au cours de la présente étude. Elles appartiennent à 51 familles. Les familles les plus représentées ont été les Caesalpiniaceae/Fabaceae avec 9 espèces à savoir: *Cassia senna, Cassia occidentalis, Cassia siamea, Cassia siebériana, Cassia alata, Cassia italica, Cassia mimosoides / Chamaecrista mimosoides, Guilandina bonduc et Piliostigma thonningii*. Les Euphorbiaceae et les Compositae ont été représentées par 8 espèces chacune suivie des Combretaceae avec 7 espèces, les Fabaceae, les Annonaceae et les Apocyanaceae avec 6 espèces chacune. Neuf familles ont été représentées par 2 espèces chacune et 32 représentées par une espèce chacune (Figure 1).

**Figure 2:**
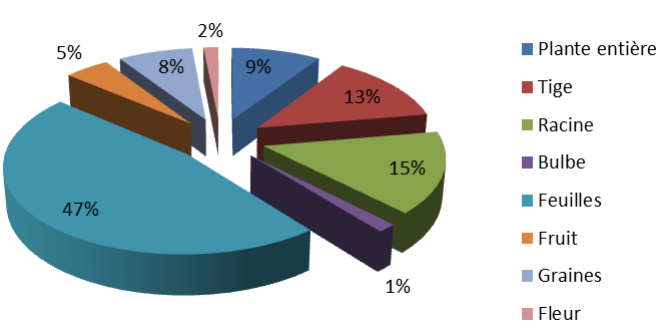
Les organes de plantes utilisés dans la préparation des recettes antidiabétiques

**Figure 3:**
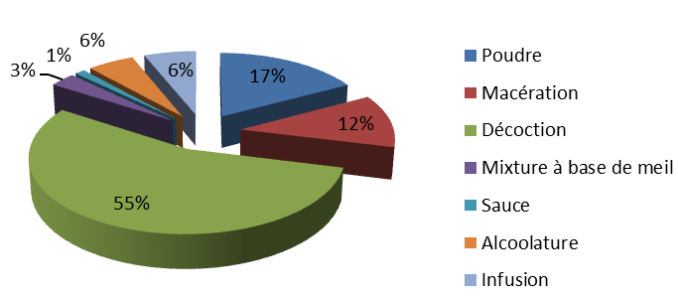
Les formes galéniques des recettes antidiabétiques

**Figure 4:**
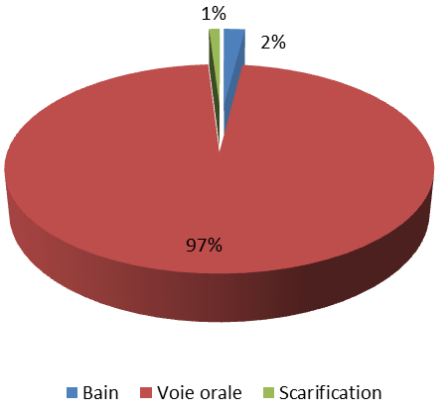
Les principales voies d'administration des recettes antidiabétiques

**Les recettes de plantes antidiabétiques**: la présente étude a permis de recenser 132 recettes préparées à partir de 112 espèces végétales dont 78 recettes à une seule plante et 54 recettes obtenues par des associations de plantes, soit respectivement 59,09% et 40,91%. Le nombre de plantes associées pour ces recettes varie entre 2 et 9. Au total, il a été recueilli 15 recettes (11,36%) à deux plantes, 18 recettes (13,64%) à trois plantes et 17 recettes (12,88%) à quatre plantes. L'importance des plantes a été matérialisée par leur valeur usuelle. Sur cette base, certaines plantes se sont révélées plus importantes que d'autres. Il s'est agi essentiellement de *Allium sativum* L. (Liliaceae), *Alium cepa* L. (Liliaceae), *Guilandina bonduc* L. (Caesalpinia/Fabaceae), *Moringa oleifera* L. (Moringaceae), *Moringa oleifera*L. (Moringaceae) et de *Picralima nitida* (Stapf) T. Durand & H. Durand (Apocynaceae) qui ont eu une valeur usuelle de 0,05 (Tableau 3, Tableau 4, Tableau 5, Tableau 6). Elles ont été suivies par un groupe de 5 plantes qui ont présenté une valeur usuelle de 0,04. Ce sont: *Catharanthus roseus* L. G. Don (Apocynaceae), *Conyza aegyptica* L. Ait.var. (Asteraceae/Compositae), *Crateva adansonii* DC. (Capparaceae), *Phyllanthus amarus* Schum. (Euphorbiaceae) et de *Xylopia aethiopica* (Dunal) A. Rich. (Annonaceae). Un deuxième paramètre utilisé pour apprécier l'importance des plantes est la citation antérieure dans les précédentes études publiées. Il est ressorti de nos résultats que 40 plantes soit environ 35% des plantes ont été cité au moins dans une étude ayant trait au diabète. Parmi les plantes citées antérieurement la plupart ont présenté des valeurs usuelles supérieures à 0,03. Cependant des plantes comme *Guilandina bonduc* (VU = 0,05), *Alium cepa* (VU = 0,05) et *Conyza aegyptica* (VU = 0,04) n'ont pas de citation antérieure. Plusieurs organes de plantes entrent dans la préparation de ces recettes antidiabétiques, tel que le montre la Figure 2. Il ressort de cette figure que les feuilles (47%) suivies des racines (15%) et des tiges (13%) sont les parties les plus utilisées. Les autres parties sont utilisées dans des fréquences inférieures à 10%. En ce qui concerne les formes galéniques, plus de 50% des recettes sont sous la forme de décoction. Les poudres et les alcoolatures sont rencontrées dans un peu plus de 10% des cas (Figure 3). Les principales voies d'administration sont la voie orale majoritairement et le bain (Figure 4).

**Tableau 3:** Les plantes et leurs modes d'emploi dans le traitement du diabète dans la région Maritime

Espèces végétales	Noms vernaculaires	N° codes	Valeur usuelle	Parties utilisées	Mode de préparation	Voies d'administration
*Acanthospermum hispidum DC. (Asteraceae/Compositae)*	Ahlungovi	746	0,01	PE	Pou	Orale
*Achyranhtes aspera I. (Amaranthaceae)*	-	-	0,01	TF	Mac	Orale
*Agelanthus dodoneifolius (DC.) Polhill & Wiens (Loranthaceae)*	-	-	0,02	Ra	Déc	Orale
*Alium cepa L. (Liliaceae)*	Sabulè	10855	0,05	Bu	Mam, Sau, Mac, Déc, Alc	Orale
*Allium sativum L. (Liliaceae)*	Ayo	10856	0,05	Bu	Mam, Sau, Mac, Déc, Alc	Orale
*Alternanthera pungens Kunth (Amaranthaceae)*	Agbaklin	1609	0,01	PE	Pou	Orale
*Anarcadium occidentale L. (Anacardiaceae)*	Yovotsan	1766	0,02	Fe, Fr	Mac, Déc	Orale
*Annona senegalensis PeRa. (Annonaceae)*	Dzogbenyikli	1877	0,01	Fe	Déc	Orale
*Annona glauca Schumach. & Thonn. (Annonaceae)*	Agnigli	1872	0,01	Fe	Déc	Orale
*Annona muricata L. (Annonaceae)*	Yévoungnigli	2267	0,01	Fe	Déc	Orale
*Anogeissus leiocarpus DC. Guill. Et Perr. (Combretaceae)*	Héhéti	476	0,01	Fe	Déc	Orale
*Anthocleista djalonensis A. Chev. (Loganiaceae/Gentianaceae)*	Gboloba	4781	0,02	Ra, Fe	Déc	Orale
*Anthocleista vogelii Planch (Loganiaceae)*	Gboloba	4781	0,01	Fe	Mac	Orale
*Azadirachta indica A. Juss. (Meliaceae)*	Kiniti	4647	0,01	Fr, G	Déc, Pou	Orale
*Blighia sapida K. D. Koenig (Sapindaceae)*	Achanti (Fisanier)	8087	0,01	Gr, Ar	Déc, Mac	Orale
*Boerhavia diffusa L. (Nyctaginaceae)*	ahozemeklo/Katson –agni	5306	0,01	PE	Pou	Orale
*Brassica oleracea L. (Brassicaceae)*	Chou commun		0,01	Fe	Mac	Orale
*Bridelia ferruginea Benth. (Euphorbiaceae)*	Akamati	3072	0,03	PE	Pou, Déc Mac,	Orale
*Bryophyllum pinnatum Lam. (Crassulaceae)*	wêkê	-	0,01	Fe	Déc	Orale
*Cannabis sativa L. (Cannabinaceae)*	Gbeku	282	0,01	Rf	Pou noire, Mac	Orale, Bain
*Carica papaya L. (Caricaceae)*	Adouba (papaya)	340	0,01	Gr, Fe	Déc, Pou	Orale
*Cassia senna Lin. (Caesalpiniaceae/Fabaceae)*	-	12	0,01	Ti	Déc	Orale
*Cassia occidentalis L. (Caesalpiniaceae/Fabaceae)*	Bessisan	115	0,01	Gr	Inf	Orale
*Cassia siamea Lam. (Caesalpiniaceae/Fabaceae)*	Zangarati-ma	121	0,01	Ra	Inf	Orale

Pou (Poudre), Déc (Décoction), Mam (Mixture au Miel), Mac (Macération), Sau (Sauce), Alc (Alcoolature), Inf (Infusion) PE (Plante Entière), TF (Tiges Feuillées), Ra (Racine), Fe (Feuille), Fr (Fruit), Gr (Graine), TE (Ecorce de Tige), Bu (Bulbe), Rf (Rameaux feuillés), Rt (Racine tubérisée), Ar (arilles), Ti (Tiges), Gs (Grains),

**Tableau 4:** Les plantes et leurs modes d'emploi dans le traitement du diabète dans la région Maritime (suite)

Espèces végétales	Noms vernaculaires	N° codes	Valeur usuelle	Parties utilisées	Mode de préparation	Voies d'administration
*Cassia siebériana DC. (Caesalpiniaceae/Fabaceae)*	Gati- gati	12521	0,01	Ra	Inf, Déc	Orale
*cassia alata L. (Caesalpiniaceae/Fabaceae)*	Dartier	-	0,01	Fe	Déc	Orale
*Cassia italica (Mill.) Lam. ex F.W.Andrews (Caesalpiniaceae/Fabaceae)*	-	-	0,01	Fe	Déc	Orale
*Cassia mimosoides / Chamaecrista mimosoides (L.) Greene (Caesalpiniaceae/Fabaceae)*	-	-	0,01	Fe	Déc	Orale
*Catharanthus roseus L. G. Don (Apocynaceae)*	Flawavigbé	2060	0,04	PE	Alc, Déc	Orale
*Ceiba pentandra (L.) Gaertn. (Bombacaceae)*	Fromager/ Kapokier	2485	0,01	Ra, TE	Déc	Orale
*Centaurea perrottetii DC. (Asteracea/Composeae)*	-	-	0,01	Fl, Ra	Déc	Orale
*Centella asiatica (L.) Urb. (Apiaceae)*	-	-	0,01	TF	Déc	Orale
*Cinnamomum zeylanicum Blume (Lauraceae)*	Canelle	-	0,01	Fe	Mac	Orale
*Cissus quadrangularis L. (Vitaceae/Ampelidaceae)*	-	-	0,01	TF	Déc	Orale
*Citrus aurantifolia Christm. Swingle. (Rutaceae)*	N'tissi	2480	0,02	Fr	Déc	Orale
*Clausena anisata (Willd.) Hook.f. ex Benth. (Rutaceae)*	Eyra	8028	0,01	Fe	Inf	Orale
*Cnestis ferruginea Vahl ex DC. (Connaraceae)*	-	-	0,01	Fe, Ra	Déc	Orale
*Coccinia grandis (L.) Voigt (Cucurbitaceae)*	-	-	0,01	Rt	Déc	Orale
*Cocoloba uvifera L. (Polygonaceae)*	Raisin de mer	-	0,01	Fe	Déc	Orale
*Coffea togoensis A. Chev (Rubiaceae)*	Coffi	7189	0,01	Gs	Pou noire, Déc	Orale
*Cola nitida (Vent.) Schott & Endl. (Sterculiaceae)*	Goro	8612	0,01	Fr	Pou noire	Orale
*Combretum glutinosum Perr. ex DC. (Combretaceae*	-	562	0,01	Fe	Déc	Orale
*Combretum micranthum G. Don (Combretaceae)*	Kinkéliba	596	0,02	Fe	Déc, Mac	Orale
*Conyza aegyptica L. Ait.var. (Asteraceae/Compositae)*	Ahlomai, Dagnigbé	869	0,04	PE	Alc, Déc	Orale
*Corchorus olitorius L. (Tiliaceae)*	Ademè	8750	0,02	Fe, G	Déc, Pou noire	Orale
*Crateva adansonii DC. (Capparaceae)*	Ouatagnyizan	326	0,04	TE, Fe	Mac, Déc, Pou	Orale
*Cymbopogon citratus Stapf. Brunel. (Poaceae)*	Tigbé	10749	0,01	Fe	Déc, Mac	Orale
*Eclipta prostrata L. (Asteraceae/Compositae)*	-	929	0,02	Fe, PE	Déc	Orale
*Ekebergia senegalensis / Ekebergia capensis Sparrm. (Meliaceae/Composeae)*	-	-	0,01	TE	Déc	Orale
*Elaeophorbia drupifera (Thonn.) Stapf (Euphorbiaceae/Meliaceae)*	-	-	0,02	Ti (sève), TE	Pou, Déc	Orale

**Tableau 5:** Les plantes et leurs modes d'emploi dans le traitement du diabète dans la région Maritime (suite)

Espèces végétales	Noms vernaculaires	N° codes	Valeur usuelle	Parties utilisées	Mode de préparation	Voies d'administration
*Erythrina senegalensis A.DC. (Fabaceae)*	Erythrine du Senegale	-	0,01	Fe	Déc	Orale
*Euphorbia hirta L. (Euphorbiaceae)*	Anossika	-	0,01	Tf	Déc	Orale
*Feretia apodanthera Delile (Rubiaceae)*	-	-	0,01	TF	Déc	Orale
*Flacourtia indica (Burm.f.) Merr. (Flacourtiaceae)*	-	-	0,01	Fe	Mac	Orale
*Gardenia ternifolia Schumach. & Thonn. (Rubiaceae)*	Kawouti/flifèti	7361	0,01	Fe	Déc	Orale
*Guiera senegalensis J.F.Gmel. (Combretaceae)*	zogbémakpavigbé	663	0,01	Fe	Déc	Orale
*Guilandina bonduc L. (Caesalpiniaceae/Fabaceae)*	Adiku	151	0,05	Fe, Ra	Déc, Mac, Pou, Mam	Orale
*Gymnema sylvestris R. BR. (Asclepiadaceae)*	-	2261	0,01	Fe	Déc	Orale
*Harrisonia abyssinica Oliv. (Simaroubaceae)*	Hedja	8461	0,02	Fe	Déc, Mac	Orale
*Holarrhena floribunda G. Don (Apocynaceae)*	Séséwou	2075	0,02	Ec, Ra	Déc	Orale
*Hygrophila auriculata (Schumach.) Heine (Acanthaceae)*	Ekpingbé/émougbé	12512	0,01	Pf	Déc	Orale
*Indigofera hiRaute L. (Fabaceae)*	Yovoviflower	-	0,01	Fe	Déc	Orale
*Irvingia gabonensis (Aubry-LeComte ex O\‘Rorke) Baill. (Irvingiaceae)*	-	-	0,01	Fe	Mac	Orale
*Jatropha curcas L. (Euphorbiaceae)*	Babatihé	-	0,02	Fe	Mac, Déc	Orale, Bain
*Jatropha gossypiifolia L. (Euphorbiaceae)*	Babatidzin	-	0,01	Gr, Fe	Déc	Orale
*Kalanchoe crenata Lam (Combretaceae)*	Aflatogan	-	0,01	Ra	Déc	Orale
*Khaya senegalensis A. Juss. (Meliaceae)*	Mahoghen	4674	0,01	Ec	Déc	Orale
*Lactuca taraxacifolia (Willd.) Schum. (Asteraceae/Compositae)*	Ahonto	1053	0,01	Fe	Inf	Orale
*Lantana camara L. (Verbenaceae)*	Fonyivi	9189	0,01	Fe	Inf	Orale
*Leea guineensis G. Don (leeaceae/Vitaceae)*	-	-	0,01	Ra	Déc	Orale
*Mallotus oppositifolius (Geiseler) Müll.Arg. (Euphorbiaceae)*	Kpamma	-	0,01	Fe	Déc	Orale
*Mangifera indica L. (Anacardiaceae)*	Mangoti	1797	0,02	Fe	Déc	Orale
*Mitragyna inermis (Willd.) Kuntze (Rubiaceae)*	Limkpati	7466	0,01	Fe, TE	Inf, Déc	Orale
*Momordica charantia L. (Cucurbitaceae)*	Agnagnra	2799	0,05	PE	Inf, Déc, Alc	Orale, Bain
*Mondia whitei (Hook.f.) Skeels (Apocyanaceae)*	Kanabo	-	0,01	Ra	Inf	Orale
*Moringa oleifera L. (Moringaceae)*	Yovoviti	5252	0,05	Gr, Ra	Mam, Alc, Pou, Déc, Pou noir	Orale, Scarification
*Ocimum basilicum L. (Lamiateae)*	Basilique centhale	4199	0,01	Fe	Déc	Orale
*Ocinum gratissimum FoRask. (Labiatae)*	Esrou	4225	0,01	Fe	Déc	Orale
*Opilia amentacea Roxb. (Opiliaceae)*	Méfiodudami/ fiodudami	5525	0,01	Fe	Pou noire	Orale

**Tableau 6:** Les plantes et leurs modes d'emploi dans le traitement du diabète dans la région Maritime (suite)

Espèces végétales	Noms vernaculaires	N° codes	Valeur usuelle	Parties utilisées	Mode de préparation	Voies d'administration
*Oxythenanthera abyssinica Munro. (Poaceae)*	Planpoti	11200	0,02	Fe	Déc, Mac	Orale
*Parkia biglobosa (Jacq.) R.Br. ex G. Don (Fabaceae/Faboideae)*	Ewoati	9468	0,01	Fe	Déc	Orale
*Phaseolus vulgaris L. (Fabaceae/Faboideae)*	Ayi	6503	0,01	Fe	Déc	Orale
*Phyllanthus amarus Schum. (Euphorbiaceae)*	Ahlivi/Ehlinvi	3367	0,04	Fe	Déc, Pou	Orale
*Picralima nitida (Stapf) T. Durand & H. Durand (Apocynaceae)*	Ayokpè	2105	0,05	PE	Mam, Alc, Déc	Orale
*Piliostigma thonningii Schum. (Caesalpiniaceae/Fabaceae)*	Klo	240	0,01	Gr	Pou	Orale
*Piper guineense Schumach. & Thonn. (Piperaceae)*	-	-	0,01	Fe	Déc	Orale
*PeRaicaria senegalensis (Meissner) Sojak (Polygonaceae)*	-	-	0,01	Fr	Déc	Orale
*Psidium guajava L. (Myrtaceae)*	Gbèbèti	10866	0,02	Fe, T, Ra	Déc, Pou	Orale
*Pterocarpus erinaceus Poir. (Fabaceae/Faboideae)*	Tem	6460	0,01	Fe	Déc	Orale
*Saba florida (Benth.) Bullock (Apocyanaceae)*	-	-	0,01	Fe + TE	Déc	Orale
*Sarcocephalus latifolius (Sm.) E.A.Bruce. (Rubiaceae)*	Nyimon	7536	0,01	Fe	Déc	Orale
*Scoparia dulcis L. (Scrophulariaceae)*	Noumayi	8440	0,01	Ra	Déc	Orale
*Securinega virosa Willd. Baill. (Euphorbiaceae)*	Hésreé	3750	0,01	PE	Déc	Orale
*Sida linifolia Juss. ex Cav. (Malvaceae)*	Odoe-ogbogbo	4488	0,01	TF	Déc	Orale
*Solanum ethiopicum L. (Solanaceae)*	Agbissan	8519	0,01	PE	Déc	Orale
*Solenostemon monostachyus (P.Beauv.) Briq. (Lamiaceae)*	-	-	0,01	Fe	Déc + Pou	Orale
*Spilanthes uliginosa/ Acmella uliginosa (Sw.) Cass. (Asteraceae)*	Flowervi	-	0,01	Fe	Déc	Orale
*Spondias monbin L. (Anacardiaceae)*	Aklicon	1853	0,02	Fe, Fl, Ra	Pou noire, Déc	Orale
*Stereospermum kunthianum Cham. (Bignoniaceae)*	-	-	0,01	Fe	Déc	Orale
*Strophantus hispidum DC (Apocyanaceae)*	-	-	0,01	Fe	Déc	Orale
*Tectona grandis L. (Verbenaceae)*	Tecti	9267	0,01	Fe	Déc	Orale
*Terminalia avicennioides Guill. & Perr. (Combretaceae)*	-	684	0,02	Fe	Pou, Déc	Orale
*Terminalia glaucescens Planch. Ex benth. (Combretaceae)*	Souwadâou	717	0,02	Fe, TE	Pou, Déc	Orale
*Theobroma cacao L. (Sterculiaceae)*	Coco	8669	0,01	Gr	Déc	Orale
*Tinospora bakis (A.Rich.) MieRa (Menispermaceae)*	-	-	0,01	Ra	Déc	Orale
*Trichilia emetica Vahl. (Meliaceae)*	Adjendjegbizou	308	0,01	Ra(écorce de)	Pou	Orale
*Uraria picta (Jacq.) DC. (Fabaceae/Faboideae)*	Vénavioda	1941	0,01	Ra (écorce de)	Déc	Orale
*Uvaria chamae P. Beauv. (Annonaceae)*	Agbana	1963	0,01	Ra	Alc, Pou Noire	Orale
*Uvariopsis guineensis Keay (Annonaceae)*	-	-	0,01	Ec, Ra	Pou	Orale
*Vernonia amygdalina Delile*	Aloma	1205	0,03	Fe	Inf, Alc, Déc, Pou noire	Orale
*Vernonia colorata Willd. Drake (Asteraceae/Compositae)*	Aloma	1208	0,01	Fe	Déc	Orale
*Xylopia aethiopica (Dunal) A. Rich. (Annonaceae)*	Etso	1987	0,04	Fr	Déc, Alc, Pou	Orale

## Discussion

La présente étude a eu pour objectif de recenser les plantes utilisées dans le traitement du diabète dans la région Maritime du Togo. Des études ethnobotaniques ont été réalisées dans la région, à l'instar de l’étude de Koudouvo et al. qui s'est penchée sur les plantes utilisées dans le traitement du paludisme [9]. En ce qui concerne le diabète, il s'agit d'une première étude du genre réalisée dans la région. L'enquête ethnobotanique a été réalisée auprès de 164 tradipraticiens de la région, qui étaient majoritairement des séniors de sexe masculin. Ce profil des tradipraticiens de la région Maritime du Togo est celui observé dans la plupart des études du genre, confirmant que la pratique de la médecine traditionnelle est l'apanage des hommes d’âge mûr [10]. Le constat établi est que la connaissance d'une recette en médecine traditionnelle est avant tout un secret de famille qui est transmis de génération en génération par le biais des coutumes et de la tradition orale. Il est donc nécessaire d'avoir un âge mature et de se faire une certaine confiance pour avoir accès aux connaissances de cette médecine. C'est la principale raison pour laquelle ce métier est pratiqué par des personnes âgées. Ce qui est d'ailleurs confirmé par le fait que les TD de la présente étude ont été dans la majorité initiés dans le cadre de la famille. La présente étude a montré une bonne diversité des plantes utilisées dans le traitement du diabète dans la région Maritime du Togo, 112 espèces végétales appartenant à 51 familles ont été recensées. D'autres études ethnobotaniques ont révélé de pareilles diversités de plantes antidiabétiques; Ziyyat et al. ont recensé 41 plantes appartenant à 36 familles [11]; Jouad et al. 54 plantes regroupées en 29 familles [12]; Tahraoui et al. 54 plantes regroupées en 25 familles [13] N'guessan et al. 19 espèces regroupées en 13 familles [14]; Adebayo 49 plantes appartenant à 33 familles [15].

Les familles les plus représentées ont été les Caesalpiniaceae/Fabaceae, les Euphorbiaceae et les Compositae. Ces résultats présentent quelques similitudes avec certains travaux antérieurs. Ainsi dans l’étude Adebayo et al., les familles les plus représentées ont été les Euphorbiaceae, les Apocynaceae, les Cucurbitaceae, les Asteraceae et les Fabaceae [15], tandis que dans l’étude de Karou et al., ce sont les Fabaceae qui ont été les plus représentées [4]. Il est ressorti de cette études que les espèces les plus utilisées par les tradipraticiens de la région Maritime pour soigner le diabète sont *Allium sativum, Alium cepa, Guilandina bonduc, Moringa oleifera* et de *Picralima nitida*. Certaines de ces plantes sont revenues dans les études effectuées par d'autres auteurs. Ainsi, dans l’étude antérieure réalisée par Adebayo au Nigeria, *Momordica charantia* et *Ocinum gratissimum* ont été les espèces les plus citées par les TD [15]. Plusieurs études ont démontré l'activité hypoglycémiante de quelques-unes des plantes citées dans notre étude, il s'agit: *Ocinum gratissimum* [16, 17]; *Momordica charantia* [18];*Phyllanthus amarus* [19]; *Allium sativum* [20]; *Aloe vera* [21]; *Psydium guajava*. Toutefois, le mode d'action de ces phytomédicaments dans l'organisme demeure mal connu. Certaines des plantes couramment revenues dans les études ethnobotaniques ont confirmé une certaine similitude entre le savoir des tradipraticiens de la région maritime du Togo et ceux des autres régions. A ce sujet, 39 sur les 112, soit 34,83% des plantes recensées ont déjà été citée dans au moins une étude publiée dans les journaux scientifiques indexés. Le reste des plantes n'a pas encore fait l'objet d'une publication en rapport avec le diabète. Parmi les plantes qui ont le plus fait l'objet d’étude scientifique en rapport avec le diabète figure en tête de liste *Momordica charantia* qui a fait l'objet d'environ une dizaine de publications dans le domaine [4, 15, 22–27]. Cette plantes est suivie de quatre autres plantes qui ont fait l'objet d'au moins cinq publications dans le domaine. Il s'agit de *Catharanthus roseus* [4, 15, 28–31] et de *Phyllantus amarus* [4, 19, 21, 32–34].

## Conclusion

Il ressort de cette étude que la région maritime du Togo dispose d'une biodiversité floristique intéressante en matière de plantes antidiabétiques. De plus les tradipraticiens de la région partagent beaucoup de similitudes dans l'utilisation des espèces végétales. Les plantes ainsi répertoriées constituent un panel qui peut servir de point de départ pour les criblages biologiques au laboratoire surtout les espèces *Guilandina bonduc, Allium cepa* et *Conyza aegyptica* qui ont une bonne valeur usuelle mais qui n'ont pas encore été citées dans les travaux scientifiques ayant abordé le traitement du diabète.
