# Corrigendum: Étude ethnobotanique des plantes utilisées dans le traitement du diabète dans la médecine traditionnelle de la région Maritime du Togo

**DOI:** 10.11604/pamj.2018.30.186.15483

**Published:** 2018-06-28

**Authors:** Holaly Efui Gbekley, Damintoti Simplice Karou, Charlemagne Gnoula, Kodjovi, Agbodeka Kokou Anani, Tchadjobo Tchacondo, Amegnona Agbonon, Komlan Batawila, Jacques Simpore

**Affiliations:** 1 Centre de Recherche et de Formation sur les Plantes Médicinales (CERFOPLAM), Université de Lomé, Togo; 2 Centre de Recherche Biomoléculaire Pietro Annigoni (CERBA), Ouagadougou, Burkina Faso

**Keywords:** Corrigendum, diabète, ethnopharmacologie, plantes médicinales, phytomédicaments, Togo, Corrigendum, diabetes, ethnopharmacology, medicinal plants, phytomedicines, Togo

## Abstract

Ce Corrigendum modifie l’article original « Étude ethnobotanique des plantes utilisées dans le traitement du diabète dans la médecine traditionnelle de la région Maritime du Togo ». The Pan African Medical Journal. 2015;20:437. doi:10.11604/pamj.2015.20.437.5660.

## Abstract (en)

This Corrgendum modifies the original article « Étude ethnobotanique des plantes utilisées dans le traitement du diabète dans la médecine traditionnelle de la région Maritime du Togo ». The Pan African Medical Journal. 2015;20:437. doi:10.11604/pamj.2015.20.437.5660

## Corrigendum

La version originale de cet article [1] contient les erreurs sur les noms des auteurs. Les corrections ont été faites dans le HTML et dans le PDF.

Les noms des auteurs: Gbekley Efui Holaly change pour Holaly Gbekley Efui. Karou Damintoti Simplice change pour Damintoti Simplice Karou. Gnoula Charlemagne change pour Charlemagne Gnoula. Agbodeka Kodjovi change pour Kodjovi Agbodeka. Anani Kokou change pour Kokou Anani. Tchacondo Tchadjobo change pour Tchadjobo Tchacondo. Agbonon Amegnona change pour Amegnona Agbonon. Batawila Komlan change pour Komlan Batawila. Simpore Jacques change pour Jacques Simpore.
